# What Is a Homœopath

**Published:** 1889-12

**Authors:** 


### WHAT IS A HOMŒOPATH?

It is interesting to note that, at the last meeting of the American Institute of Homœopathy, which was held at Lake Minnetonka, Minn., a proposed amendment to the by-laws, by which future applicants for membership should be required to be "Believers in, and practitioners of Homœopathy," was defeated by seventy-six votes to thirty-four! This is a very curious commentary upon the pretensions of those practitioners of medicine who adhere to this trade-mark. There are some things which a proper exercise of charity, and a due regard for the right of individual judgment, require of the candid critic; but it is straining charity a little too much to expect any candid critic—who is not, at the same time, a very timid one—to condone such inconsistency as is displayed when the representative body of homœopaths declines to require of its members that they shall believe and practice what they profess.

In strange contrast to this is the dictum of Hahnemann: "Away with false doctors, who profess to be preservers of human life, but whose heads are filled with vain deceit!" It is curious, in reading the homœopathic journals published in this country, to see how they dodge around occurrences of this kind, and how entirely they fail to appreciate the moral aspect which they present to those who believe that the same principle should rule in medicine as rules in every other department of science or art.—*Boston Journal of Health.*

